# Intake of *trans*-fats among US youth declined from 1999–2000 to 2009–2010

**DOI:** 10.1017/S1368980019003367

**Published:** 2020-04

**Authors:** Brandon J Restrepo

**Affiliations:** US Department of Agriculture, Economic Research Service, 355 E. Street SW, Washington, DC 20024, USA

**Keywords:** *Trans*-fatty acids, CVD, National Health and Nutrition Examination Survey, Children, Adolescents, USA

## Abstract

**Objective:**

To analyse how much the intake of *trans*-fatty acids (TFA), an important dietary risk factor for CVD, changed among US children and adolescents over a period of time when food regulations that reduced the presence of TFA in the food supply were enacted.

**Design:**

Regression models were used to estimate changes in levels of TFA in plasma and other CVD risk factors among US children and adolescents from 1999–2000 to 2009–2010.

**Setting:**

USA.

**Participants:**

Nationally representative sample of children (aged 6–11 years) and adolescents (aged 12–19 years) who participated in the 1999–2000 and 2009–2010 cycles of the National Health and Nutrition Examination Survey.

**Results:**

Levels of plasma TFA declined significantly by an average of 61·9 % from 1999–2000 to 2009–2010. The average decline in a TFA commonly found in partially hydrogenated oils (elaidic acid, 67·2 %) was larger than the average decline in a TFA naturally occurring in ruminant animals (vaccenic acid, 60·5 %). Significant improvements in a variety of other CVD risk factors (LDL- and HDL-cholesterol, TAG, systolic and diastolic blood pressure, C-reactive protein) were also observed.

**Conclusions:**

Between the two time points, 1999–2000 and 2009–2010, there were substantial decreases in plasma TFA levels and improvements in several other important CVD risk factors in the population of US children and adolescents.

Consumption of *trans*-fatty acids (TFA) is a well-known risk factor for CVD such as heart disease^(1)^. Observational studies suggest that a 4·184 kJ (1 kcal) increase in TFA intake increases the risk of heart disease by a larger amount than does the same-sized increase in the intake of any other nutrient^(2)^. Heart disease is a major cause of death in the USA^(3,4)^, so it is important to know how this dietary CVD risk factor has changed over time, especially for vulnerable sub-populations over a period of time when food regulations that reduced the presence of TFA in the food supply were enacted. Prior research has shown that average levels of plasma TFA among US adults fell by over 50 % between 1999 and 2010^(5,6)^, but previous data limitations did not allow for an analysis of the amount by which TFA levels declined among younger individuals. While children and adolescents are at a lower risk of developing CVD than adults, intake of TFA in early childhood and adolescence could set in motion processes that cause CVD in adulthood^(7)^.

Using data that were released in March 2019 from the 1999–2000 and 2009–2010 National Health and Nutrition Examination Survey (NHANES), the present study contributes to the literature by analysing differences over time in levels of plasma TFA in a nationally representative sample of children and adolescents. Since TFA consumption raises the risk of CVD in part by lowering HDL-cholesterol (HDL-C), raising LDL-cholesterol (LDL-C) and promoting systemic inflammation^(8)^, a complementary analysis of differences over time in a variety of other important CVD risk factors is also conducted.

## Methods

**Fig. 1:**
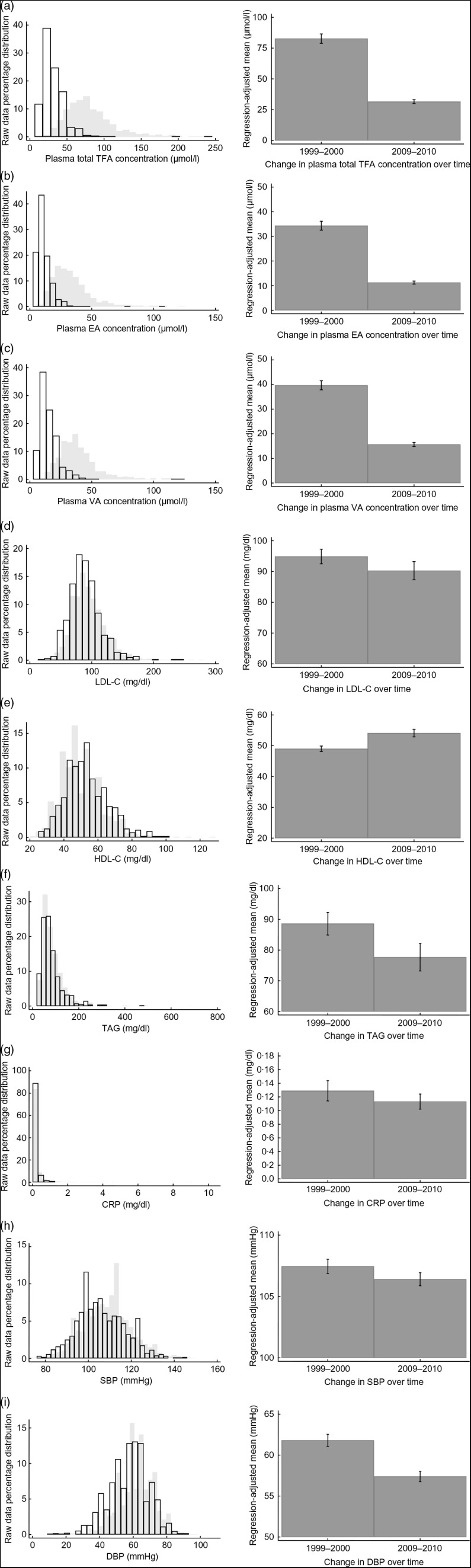
Distributions and regression-adjusted means of plasma *trans*-fatty acids (TFA) and other CVD risk factors in 1999–2000 and 2009–2010 among US children (aged 6–11 years) and adolescents (aged 12–19 years) who participated in the 1999–2000 and 2009–2010 cycles of the National Health and Nutrition Examination Survey. Each panel presents results pertaining to a different dependent variable: (a) total TFA* (*n* 1126); (b) elaidic acid (EA; *n* 1170); (c) vaccenic acid (VA; *n* 1173); (d) LDL-cholesterol (LDL-C; *n* 1828); (e) HDL-cholesterol (HDL-C; *n* 1836); (f) TAG (*n* 1837); (g) C-reactive protein (CRP; *n* 5152); (h) systolic blood pressure (SBP; *n* 4536); (i) diastolic blood pressure (DBP; *n* 4457). The left column shows the distributions of the raw data (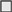, 1999–2000; 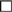, 2009–2010) and the right column shows the change in each dependent variable over time expressed as regression-adjusted means with 95 % confidence intervals represented by vertical bars. Fasting sampling weights were used in regressions involving the dependent variables total TFA, EA, VA, LDL-C, HDL-C and TAG. Examination sampling weights were used in regressions involving the dependent variables CRP, SBP and DBP. *Total TFA = sum of EA, VA, linoelaidic acid and palmitelaidic acid

The NHANES assesses the health and nutritional status of Americans through a combination of interviews, laboratory tests and physical examinations, and is designed to be representative of the US civilian non-institutionalized population. Children (6–11 years of age) and adolescents (12–19 years of age) in the 1999–2000 and 2009–2010 cycles of the NHANES with information on the following variables were selected for the analysis: four TFA (elaidic, vaccenic, linoelaidic and palmitelaidic acids); C-reactive protein; LDL-C and HDL-C; TAG; and systolic and diastolic blood pressure. The sum of the four TFA was considered in the analysis and two types were analysed in isolation: (i) elaidic acid, which is commonly found in partially hydrogenated oils (PHO); and (ii) vaccenic acid, which is naturally occurring in ruminant animals and present in small quantities in dairy products, but is also present in industrially produced TFA. While the number of observations available for the regression analysis varies by the dependent variable under consideration (see sample sizes in Fig. 1 caption), the average demographic profile (age, sex and race/ethnicity) is similar across regression samples. Information on TFA, LDL-C, HDL-C and TAG measures is available only for those in the fasting morning session while information on the other measures is available for the full sample. Data on all measures for individuals aged 6–19 years are available in both cycles, except for systolic and diastolic blood pressure, which are available only for those aged 8–19 years. Data on TFA for children and adolescents were publicly released in March 2019. All analyses summarized in the present study were conducted in July 2019 using the statistical software package Stata version 15.1.

To estimate differences in TFA levels and other CVD risk markers between cycles, the following regression model was estimated by ordinary least squares:

(1)

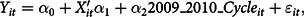

where *Y*_*it*_ is a CVD risk factor for individual *i* at time *t*; *X*_*it*_ is a vector of individual characteristics (age, sex and race/ethnicity) that accounts for changing demographic characteristics over time that could affect CVD risk factors in the population of children and adolescents; 2009_2010_*Cycle*_*it*_ is an indicator variable equal to 1 for the 2009–2010 cycle and 0 for the 1999–2000 cycle; and *ε_it_* is a random error term. There was significant skewness in the dependent variables, so they were log-transformed in the regression analysis. Robust standard errors were estimated using the appropriate sampling weights, strata and primary sampling units to account for the complex survey design of the NHANES.

After the regression model was estimated, the regression coefficient estimates were used to calculate predicted values for the CVD risk factors, one for the 1999–2000 cycle ( 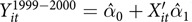 ) and another for the 2009–2010 cycle ( 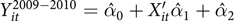 ), where 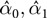 and 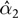 are the estimated regression coefficients from equation (1). In the results below, we present regression-adjusted mean values for the 1999–2000 and 2009–2010 cycles – i.e. mean values of 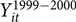 and 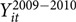 , respectively – in their original units for ease of interpretation.

## Results

Figure 1 shows the distributions of raw data (left column) and regression-adjusted means (right column) by dependent variable. The distributions of CVD risk factors – especially plasma TFA concentrations – show shifts to the left from 1999–2000 to 2009–2010. The exception is HDL-C, which shows a shift to the right. On average levels of plasma TFA declined significantly from 82·69 to 31·53 µmol/l or by an average of 61·9 % from 1999–2000 to 2009–2010 (see right column of panel (a), Fig. 1). Levels of elaidic acid fell by a larger amount (from 34·33 to 11·25 µmol/l) than did those of vaccenic acid (from 39·67 to 15·66 µmol/l), which translate into declines of 67·2 and 60·5 %, respectively (see right column of panels (b) and (c), Fig. 1). When equations involving each of these acids were estimated in a seemingly unrelated regression framework, the results indicated that these changes are significantly different from each other (*P* < 0·01).

Significant improvements in other CVD risk factors from 1999–2000 to 2009–2010 among youth were also observed (see right column of panels (d) to (i), Fig. 1). The results indicate that on average their lipid profile improved, with LDL-C falling by 4·9 %, HDL-C rising by 10·4 % and TAG falling by 12·3 %. Turning to an important marker of inflammation in the body (C-reactive protein), the analysis also indicates that its average levels also fell by 12·3 %. And finally, average blood pressure readings improved as well, with systolic and diastolic blood pressure falling by 1·0 and 7·1 %, respectively.

## Discussion

From 1999 to 2010, significant declines in levels of plasma TFA were observed in the US population of children and adolescents. A complementary analysis revealed significant improvements in a host of other important CVD risk factors as well, some of which have been causally linked to TFA consumption (e.g. LDL-C, HDL-C and C-reactive protein)^(8)^. Taken together, as has been found for US adults^(5,6)^, the evidence reported here shows that several dimensions of the cardiovascular health of US children and adolescents also improved between 1999 and 2010.

Over the study period, two major food policy initiatives were implemented with the goal of reducing population-level intake of TFA. Starting in 2006, the Food and Drug Administration requires food manufacturers to declare TFA content on the Nutrition Facts label of packaged foods and shortly afterwards several localities and states started restricting the use of PHO in food-service establishments, with the percentage of the US population living in a jurisdiction with a PHO restriction growing from an estimated 3 to 20 % between 2007 and 2010^(9)^. It is possible that these food regulations contributed to improvements in the CVD risk factors observed in the present study^(10–12)^. The larger decline over time in a TFA commonly found in PHO may be viewed as supportive of this interpretation since PHO are the principal source of artificial or industrially produced TFA, which are easier to eliminate from the food supply than are ruminant TFA^(13)^. Voluntary responses by national food producers to rising demand for healthier foods, such as the phasing out of the use of PHO in foods by Dunkin’ Donuts, Burger King, McDonald’s and other national food chains in the mid-to-late 2000s, may have also played a role in the improvements in the cardiovascular health profile of youth in the USA observed here^(14)^.

### Limitations

Despite the strengths of the present study, particularly the use of biomarker data on a host of important CVD risk factors, the analysis was limited to only two cycles of the NHANES that are separated by a decade and thus it cannot determine the causes of the differences in the outcomes analysed. There were policy changes over the study period that did not target TFA consumption (e.g. anti-smoking regulations), which may have also contributed to improvements in some of the CVD risk factors analysed here. An important direction for future research is to pinpoint and compare the causal impacts of a variety of policy actions on industrially produced TFA and CVD in the population, such as the ones contained in the WHO’s REPLACE action plan to eliminate artificial *trans*-fat in the global diet^(15)^. Such a research endeavour may help to inform policy-making decisions regarding potential actions to reduce TFA consumption in other countries that are considering regulation of TFA in the food supply in an effort to improve cardiovascular health at the population level^(16)^.

## Conclusions

Similar to what previous research has shown for adults^(5,6)^, the present study finds that levels of plasma TFA in a nationally representative sample of US children adolescents also fell substantially between 1999 and 2010. There were also significant improvements in several other important CVD risk factors. In 2011, it was estimated that CVD was responsible for about one in every six health-care dollars nationally and medical costs of CVD are projected to rise between 2010 and 2030^(17)^. As of June 2018, for the majority of uses of PHO, the Food and Drug Administration prohibits food manufacturers from adding PHO to foods. Assuming improvements in the CVD risk factors between 1999 and 2010 are at least partly attributable to previously enacted food regulations that reduced the presence of TFA in the food supply, the Food and Drug Administration’s recently implemented nationwide ban on TFA may help to mitigate the projected rise in the economic burden of CVD.
